# Dexmedetomidine exerts dual effects on human annulus fibrosus chondrocytes depending on the oxidative stress status

**DOI:** 10.1042/BSR20190419

**Published:** 2019-08-23

**Authors:** Lin Zhou, Jinhua Zhou, Bin Sheng, Xin Li, Youchao Yuan

**Affiliations:** 1 Department of Clinical Laboratory, Hunan Provincial People’s Hospital, The First Hospital of Hunan Normal University, No. 61 Jiefang West Road, Changsha 410000, Hunan, China; 2 Department of Orthopedics, Hunan Provincial People’s Hospital, The First Hospital of Hunan Normal Universtiy, No. 61 Jiefang West Road, Changsha 410000, Hunan, China

**Keywords:** annulus fibrosus chondrocytes, cartilage degeneration, Dexmedetomidine, NF-κB, NLRP3, XIAP

## Abstract

Dexmedetomidine (Dex) is an anesthetic widely used in lumbar discectomy, but its effect on chondrocytes remains unclear. Dex is speculated to promote cartilage degeneration by activating α-2 adrenergic receptor. However, the antioxidative and anti-inflammatory effects of Dex implied the potential chondrocyte protective effect under stress conditions. The present study aimed to determine the effect of Dex on chondrocytes under non-stress and stress conditions. Chondrocytes were isolated from human annulus fibrosus (AF) tissues and oxidative stress was induced by treatment with 1 mM hydrogen peroxide (H_2_O_2_). Chondrocytes were treated with Dex alone or in combination with H_2_O_2_. Treatment with Dex alone decreased mRNA expression of *COL2A1* and increased that of *MMP-3* and *MMP-13*, thus contributing to cartilage degeneration. However, Dex prevented H_2_O_2_-induced death and degeneration of chondrocytes partly by enhancing antioxidant capacity. Mechanistically, Dex attenuated H_2_O_2_-mediated activation of NF-κB and NACHT, LRR, and PYD domains-containing protein 3 (NLRP3), both of which play key roles in inflammation and inflammatory damage. Dex inactivated NLRP3 through the suppression of NF-κB and JNK signals. Co-treatment with Dex and H_2_O_2_ increased protein level of XIAP (X-linked inhibitor-of-apoptosis, an anti-apoptosis protein), compared with H_2_O_2_ treatment alone. H_2_O_2_ treatment increased the expression of neural precursor cell expressed developmentally down-regulated protein 4 (NEDD4) that is a ubiquitin ligase targeting XIAP. However, Dex decreased the amount of NEDD4 adhering to XIAP, thus protecting XIAP protein from NEDD4-mediated ubiquitination and degradation. Given that surgery inevitably causes oxidative stress and inflammation, the protective effect of Dex on chondrocytes during oxidative stress is noteworthy and warrants further study.

## Introduction

The intervertebral disc (IVD) is the largest avascular organ in the human body, and metabolic exchange is predominantly reliant on the diffusion effect across cartilage endplate. Due to these structural and metabolic characteristics, chondrocytes within the IVD are sensitive to multiple etiological factors including aging, smoking, infection, abnormal mechanical stress, diabetes, trauma, and genetic predisposition [1]. Accumulative evidence indicates that overproduction of reactive oxygen species (ROS), which results in oxidative stress, plays a critical role in the damage of chondrocytes upon exposure to these etiological factors [1]. Excessive ROS reinforces inflammation and degradation of the extracellular matrix, changes cartilaginous phenotype, accelerates senescence of disc cells, and even triggers programmed cell death [2,3]. These pathological reactions are dependent on ROS acting as crucial intermediary factors in the signaling network and on the strong oxidation characteristics. These pathological changes consequently reduce the ability of IVD to bear weight causing several pathophysiological features, such as lower back pain (LBP), sciatica, and cauda equina symptoms. Each year in China, LBP affects millions of people, especially those over 50 years of age, which largely increases the social burden.

The adrenoreceptor (AR) family of proteins comprises α1, α2, and β subtypes and are constitutively expressed in chondrocytes. It has been identified that three subtypes of ARs are involved in the modulation of various physiological and pathological actions of chondrocytes. α1-AR mediates norepinephrine (NE)-induced activation of caspase-3 and 7 and subsequent apoptosis of chondrocytes, since α1-AR inhibitor, but not inhibitors of α2- and β-AR, abolished the apoptotic effect of NE [4]. Activation of α2-AR by NE decreases the expression of aggrecans but stimulates the production of MMP-3, MMP-13, and RANKL by chondrocytes, which suggests the potential role of α2-AR in cartilage degeneration [5]. However, these effects of α2-AR are dependent on the activation of the ERK1/2 and PKA pathways [5]. It is known that these signaling cascades are critical for cell proliferation and survival. Stimulation of β(2)-AR by isoproterenol inhibited mRNA and protein levels of Col II and Sox-6 by activating ERK1/2 and PKA [6]. Activation of β-AR by NE slows down the cell cycle and decreases cell proliferation [4]. Nevertheless, NE reverses IL-1β-induced gene expression of IL-8, MMP-13, glycosaminoglycan, and collagen II via β-AR activation [4], thereby exhibiting an anti-inflammatory effect.

Dexmedetomidine (Dex), a selective agonist of α2-AR, has been increasingly used during or after surgery for its sedative, analgesic, and sympatholytic effects. Theoretically, the activation of α2-AR by Dex likely induces cartilage degeneration by modulating the expression of aggrecans, MMP-3, MMP-13, and RANKL. However, substantial data indicate that Dex confers strong protection against cell damage caused by oxidative stress, inflammation, and hypoxia [7–9]. These protective effects of Dex are primarily due to its regulation of signaling molecules such as NACHT, LRR, and PYD domains-containing protein 3 (NLRP3), Nrf-2, NF-κB, ERK1/2, and PKA through the activation of α2-AR [7–9]. Since the protective effects of Dex has been reported in neurocytes, nephrocytes, and cardiomyocytes, we hypothesized that Dex could also exert protective effect on chondrocytes, especially when they are exposed to ROS. ROS are the key inducers of degeneration and death of chondrocytes. The effect of Dex against ROS is supposed to hinder this phenomenon. Thus, it is likely that Dex plays different roles in the degeneration of chondrocytes under non-stress and stress conditions.

Lumbar discectomy is a common surgical procedure for the management of LBP resulting from IVD degeneration, but this surgery inevitably causes tissue damage and thus triggers oxidative stress and inflammation. Given that Dex is widely used in lumbar discectomy to date, it is worthwhile to investigate the effect of Dex on chondrocytes under stress conditions. The present study aimed to determine the effect of Dex on IVD chondrocytes against oxidative stress induced by hydrogen peroxide (H_2_O_2_) and the underlying mechanism of Dex.

## Materials and methods

### Isolation and culture of human chondrocytes

Patients [four males and four females, mean age  =  56 years (age: 43–72 years)] with LBP caused by disc herniation in the lumbar region, were enrolled in the present study. All participants provided written informed consent. The present study was approved by the Local Ethics Committee of Hunan People’s Hospital (Changsha, People’s Republic of China). These patients underwent lumbar discectomy by epiduroscopy, which is a minimally invasive technique, in Hunan People’s Hospital between January 2017 and March 2018. Human chondrocytes were isolated from annulus fibrosus (AF) tissues that were obtained from lumbar discectomy. The AF tissues were minced into small pieces (1 mm^3^) and digested with 0.25% trypsin for 30 min at 37°C. After three washes with PBS, the samples were further treated with 0.2% collagenase type II for 4 h at 37°C. Isolated chondrocytes were passed through a 70-mm nylon mesh (Falcon) to remove residual cartilage matrix fragments. The isolated cells were cultured in complete culture medium (DMEM/F12, Life Technologies, Carlsbad, CA, U.S.A.) supplemented with 10% fetal bovine serum (FBS, Invitrogen, Carlsbad, CA, U.S.A.) and 1% penicillin/streptomycin (Life Technologies) in a humidified 5% CO_2_ incubator at 37°C.

### Immunocytochemistry assay

Chondrocytes were cultured on coverslips in six-well plates. Cells were fixed with 4% paraformaldehyde for 10 min at room temperature and then with methanol at −20°C for 20 min. Normal goat serum (10%, HyClone, GE Healthcare Life Sciences, Shanghai, China) was added to the cells for 30 min to block non-specific binding sites. The fixed cells were immunostained with primary antibodies targeting collagen II (dilution 1:800, Abcam, Cambridge, United Kingdom) overnight at 4°C followed by biotin–conjugated secondary antibody (1:500 dilution, ZSGB-BIO, Beijing, China) for 1 h at 37°C. Finally, sections were lightly counterstained using Hematoxylin at 37°C for 1 min. Images were acquired using a high-resolution CoolSNAP™ CCD camera (Photometrics Inc., Tucson, AZ, U.S.A.) under the control of a computer using Leica FW4000 software, version 1.2 (Leica Microsystems, Ltd., Milton Keynes, U.K.).

### Cell treatments

The chondrocytes were cultured in DMEM with 2% FBS for 12 h before and during the following treatments. Chondrocytes were treated with doses of Dex for different time periods to determine the optimum concentration and culture time. Oxidative stress was induced by treating the cells with 1 mM H_2_O_2_ (Sigma–Aldrich Chemical Co., U.S.A.) for 1 h. Afterward, cells were cultured in fresh medium or the medium with the supplementation of Dex (5 μM, Sigma Chemicals, St. Louis, MO, U.S.A.), tomatidine (10 μM, NF-κB inhibitor, Selleck, Shanghai, China), SP600125 (5 μM, JNK inhibitor, Selleck), or CY-09 (1 μM, NLRP3 inhibitor, Selleck) with further incubation for 24 h. The cells were washed twice after H_2_O_2_ treatment to avoid the potential chemical reaction between H_2_O_2_ and other agents. Cells in the control group received no above treatments.

### Cell viability assay

Cells were seeded in 96-well plates (3 × 10^6^ cells per well) for 24 h. After the cell treatments, cell viability was detected using cell counting kit-8 (CCK-8, Dojindo, Kyushu, Japan) according to the manufacturer’s instructions.

### Flow cytometry analysis

Cell death rate was detected by flow cytometry. Cells were seeded in six-well plates. Apoptotic incidence was analyzed by the Annexin V-FITC/ Propidium Iodide (PI) detection kit (Beyotime Institute of Biotechnology, Shanghai, China) according to the manufacturer’s instructions. Briefly, cells were stained with Annexin V-FITC and PI for 15 min in the dark at room temperature. The rate of apoptosis was analyzed using a dual laser flow cytometer (Becton Dickinson; San Jose, CA, U.S.A.) and estimated using the ModFit LT software, version 1.0 (Verity Software House; Topsham, ME, U.S.A.).

### Measurement of intracellular ROS levels

The production of intracellular ROS was monitored using a cell-permeable fluorogenic probe, 2′,7′-dichlorodihydrofluorescein diacetate (H2DCFDA, Invitrogen). After the above indicated treatments, the cells were harvested and stained with 10 μM H2DCFDA (Sigma–Aldrich Chemical Co.) in the dark at 37°C for 15 min. The cells were then rinsed twice with PBS, and 10000 events were immediately analyzed using a flow cytometer (Becton Dickinson) with an excitation wavelength of 480 nm and an emission wavelength of 525 nm.

### Antioxidant enzyme activity assay

The activities of antioxidant enzymes including catalase (CAT), superoxide dismutase (SOD), and glutathione peroxidase (GPx) were estimated by using the commercially available assay kits from Beyotime Bio-Corporation (Shanghai, China) as per the manufacturer’s instructions. To analyze the activity of CAT, cell homogenates were taken in a cuvette containing H_2_O_2_ at a known concentration and was catalyzed by CAT in our samples for an exact time between 1 and 5 min. The remaining H_2_O_2_ was coupled with a substrate and treated with peroxidase to generate a red product, N-4-antipyryl-3-chloro-5-sulfonate-*p*-benzoquinonemonoimine, which absorbs maximally at 520 nm. By estimating the remaining H_2_O_2_, we can calculate the amount of H_2_O_2_ that reacted with CAT and finally determine CAT activity. SOD present in our samples inhibited the process of superoxide transforming WST-8, a 2-(4-iodophenyl)-3-(4-nitrophenyl)-5-(2,4-disulfophenyl)-2H-tetrazolium monosodium salt, to a stable water-soluble WST-8 formazan. The latter can be evaluated by testing the optical density at 450 nm whereby SOD activity can be determined. The determination of GPx activity was based on the principle that nicotinamide adenine dinucleotide phosphate (NADPH) continually diminishes in the cycle of GPx transforming reduced glutathione to oxidized glutathione that is returned to reduced glutathione by glutathione reductase. Detecting reduced NADPH at an absorbance of 340 nm can indirectly estimate GPx activity.

### RT-qPCR analysis

RNA was extracted using TRIzol/chloroform (15596-018, Invitrogen, Carlsbad, CA, U.S.A.) according to the manufacturer’s instructions. cDNA (1 μg) was reverse-transcribed from RNA using a reverse transcription kit (Applied Biosystems, Foster City, CA, U.S.A.) and mixed with the primers (Table 1) and Fast Universal Master Mix (Applied Biosystems). Gene expression was examined by RT-qPCR. Data were analyzed by the comparative 2^−ΔΔ*C*^_t_ method, with the housekeeping gene *GAPDH* as an internal control. Results were presented as gene expression relative to control (fold change).

**Table 1:** Primers used in PCR assay

Name		Sequence (5′–3′)	Tm (°C)	Amplicon size (bp)
Aggrecan	Forward	ACTCTGGGTTTTCGTGACTCT	61	81
	Reverse	ACACTCAGCGAGTTGTCATGG		
Col2A1	Forward	TGGACGCCATGAAGGTTTTCT	62	183
	Reverse	TGGGAGCCAGATTGTCATCTC		
MMP-3	Forward	CTGGACTCCGACACTCTGGA	62	79
	Reverse	CAGGAAAGGTTCTGAAGTGACC		
MMP-13	Forward	ACTGAGAGGCTCCGAGAAATG	61	103
	Reverse	GAACCCCGCATCTTGGCTT		
ADAMTS1	Forward	TTCCACGGCAGTGGTCTAAAG	62	100
	Reverse	CCACCAGGCTAACTGAATTACG		
NLRP3	Forward	GATCTTCGCTGCGATCAACAG	61	81
	Reverse	CGTGCATTATCTGAACCCCAC		
XIAP	Forward	AATAGTGCCACGCAGTCTACA	61	103
	Reverse	CAGATGGCCTGTCTAAGGCAA		
GAPDH	Forward	GGAGCGAGATCCCTCCAAAAT	61	197
	Reverse	GGCTGTTGTCATACTTCTCATGG		
miR-223-3p	Forward	UGUCAGUUUGUCAAAUACCCCA	61	
	Reverse	CAGTGCGTGTCGTGGAGT		
miR-302-3p	Forward	TAAGTGCTTCCATGTTTTGGTGA	61	
	Reverse	CAGTGCGTGTCGTGGAGT		
miR-520-3p	Forward	ACACTCCAGCTGGGAAAGTGCTTCCC	61	
	Reverse	CTCAACTGGTGTCGTGGA		
U6	Forward	GCTTCGGCAGCACATATA	61	
	Reverse	AACGCTTCACGAATTTGCGT		

Abbreviation: XIAP, X-linked inhibitor-of-apoptosis.

### Western blot assay

Western blot assay was performed to examine the expression levels of indicated proteins in AF chondrocytes. Protein extracts were separated using 10–12% SDS/polyacrylamide gel electrophoresis and transferred on to nitrocellulose membranes. The membranes were incubated with the following primary antibodies, phospho (p)-p65 (1:1000; ab76302, Abcam), NLRP3 (1:500; ab214185, Abcam), caspase-1 (1:500; ab207802, Abcam), IL-1β (1:500; ab9722, Abcam), X-linked inhibitor-of-apoptosis (XIAP; 1:500; ab28151, Abcam), neural precursor cell expressed developmentally down-regulated protein 4 (NEDD4; 1:500; ab46521, Abcam), and GAPDH (1:1000; ab181602, Abcam) at 4°C overnight. The primary antibodies were visualized by adding biotin–conjugated secondary antibodies followed by an avidin/biotin/peroxidase enzyme complex (Vectastain ABC Elite kit; Vector Laboratories Inc, Burlingame, CA, U.S.A.) and an appropriate substrate (Vector Nova RED, Vectastain).

### Immunoprecipitation assay

Cells were lysed with immunoprecipitation assay lysis buffer (RIPA, Sigma–Aldrich). Cell lysates with equal amount of protein (500 μg) were incubated with nickel beads conjugated to anti-XIAP antibody (Abcam) for 3 h, followed by washing with IP buffer (50 mM Tris, pH 7.5, 5 mM EDTA, 150 mM NaCl, and 0.5% NP-40). Bound proteins were detected by Western blotting using primary antibody against NEDD4 (Abcam) and HRP–conjugated secondary antibody.

### Statistical analysis

Results were presented as mean ± standard deviation. Statistical analysis was performed using SPSS software, version 11.0 (SPSS, Chicago, IL, U.S.A.). One-way analysis of variance (ANOVA) was used for data analysis, followed by least significant difference test (Fisher’s test) and the unpaired Student’s *t* test was used for comparison between two means. *P*-values less than 0.05 (*P*>0.05) were considered as statistically significant.

## Results

### Dex improved the proliferation of AF chondrocytes in the presence or absence of H_2_O_2_

**Figure 1:**
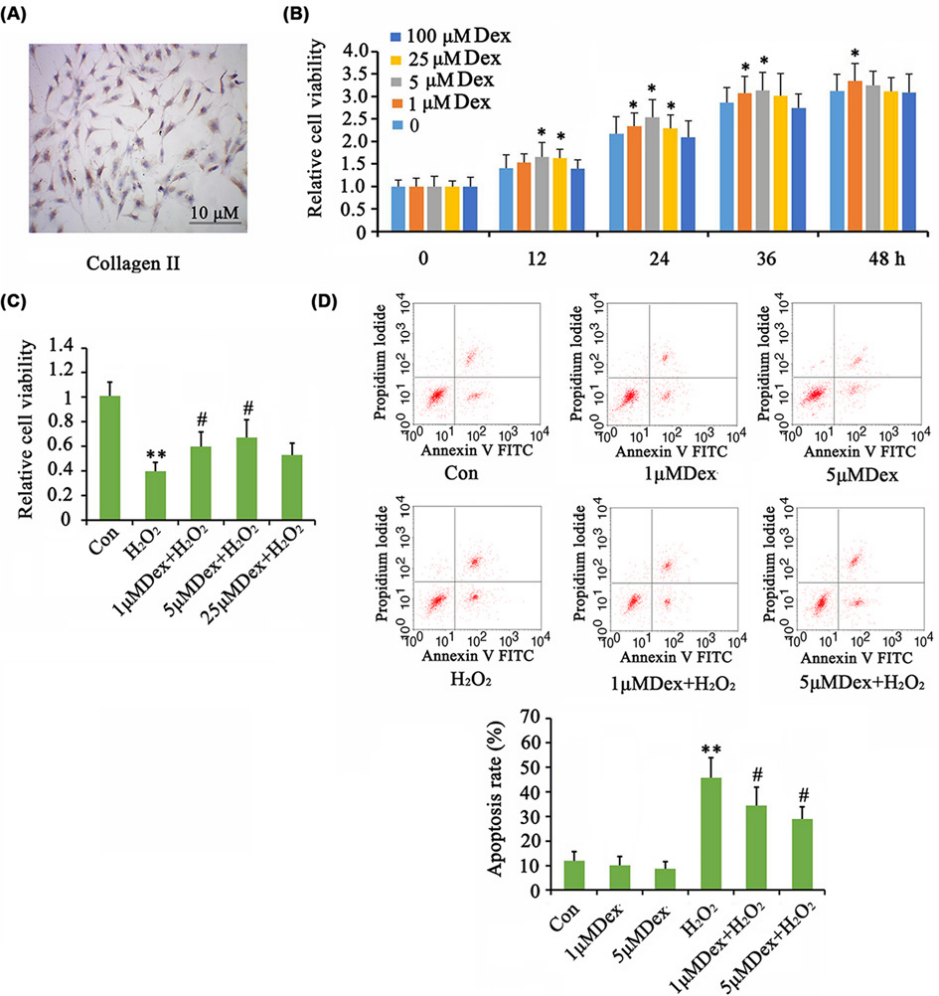
Dex improved the proliferation of AF chondrocytes in the presence or absence of H_2_O_2_ The isolated AF chondrocytes were identified by ICC assay (**A**). Chondrocytes were treated with doses of Dex for different time periods to determine the optimum concentration and culture time. Then cell viability was tested (**B**). Oxidative stress was induced by treating the cells with 1 mM H_2_O_2_ for 1 h. Afterward, cells were cultured in fresh medium or the medium with the supplementation of Dex with further incubation for 24 h. Then cell viability (**C**) and apoptosis rate (**D**) were tested. *P<0.05 and **P<0.01 *vs.* control group; ^#^*P*<0.05 *vs.* the group that was treated with H_2_O_2_ alone.

The isolated AF chondrocytes were identified by immunocytochemistry (ICC) assay (Figure 1A). The cells were stained with collagen II antibody–conjugated dye, but the staining was relatively weak suggesting some degree of degradation of the chondrocytes. As determined by CCK-8 assay, the viability of AF chondrocytes was increased by Dex at doses of 1, 5, and 25 μM at 12, 24, 36, or 48 h (*P*<0.05, Figure 1B). Treatment with 5 μM Dex showed a better effect on cell viability than at other dosages at 24 and 36 h. Cell viability was notably decreased by H_2_O_2_ (*P*<0.01, Figure 1C), but treatment with 1 and 5 μM Dex significantly reversed cell viability (*P*<0.05 vs. H_2_O_2_ group). Flow cytometry analysis showed that the death rate of AF chondrocytes was not significantly changed by Dex at the tested range of concentrations (Figure 1D). A dramatic increase in the rate of cell death was observed after H_2_O_2_ treatment (*P*<0.01). Treatment with 1 and 5 μM Dex lowered the death rate with more profound effect at 5 μM Dex (*P*<0.05 vs. H_2_O_2_ group).

### Dex affected the degeneration of AF chondrocytes

**Figure 2:**
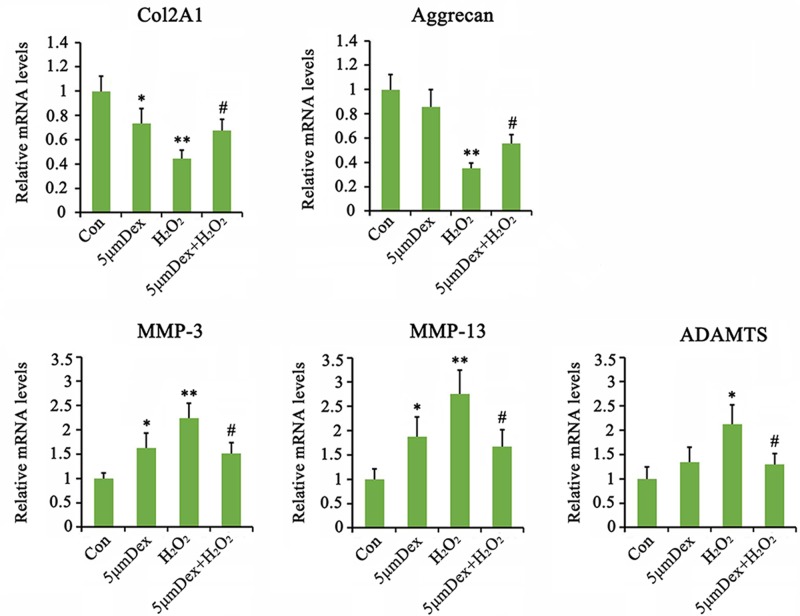
Dex regulated the degeneration of AF chondrocytes Chondrocytes were treated with 5 μM Dex for 24 h. In addition, chondrocytes were treated with 1 mM H_2_O_2_ for 1 h. Afterward, cells were cultured in fresh medium or the medium with supplementation of 5 μM Dex with further incubation for 24 h. The relative expression levels of indicated mRNA were tested by PCR. *P<0.05 and **P<0.01 *vs.* control group; ^#^*P*<0.05 *vs.* the group that was treated with H_2_O_2_ alone.

The mRNA expression of *COL2A1*, but not aggrecan, was decreased with Dex treatment (*P*<0.05, Figure 2). Dex conversely increased the expression of *MMP-3* and *MMP-13* (*P*<0.05), but not *ADAMTS5*. Stimulation by H_2_O_2_ caused the dramatic reduction in *COL2A1* (*P*<0.01) and aggrecan (*P*<0.01) expression and increase in *MMP-3* (*P*<0.01), *MMP-13* (*P*<0.01), and *ADAMTS* (*P*<0.05) expression in AF chondrocytes. Treatment with Dex after H_2_O_2_ blocked the reduction in *COL2A1* and aggrecan as well as the increase in *MMP-3, MMP-13*, and *ADAMTS* expression (*P*<0.05 vs. H_2_O_2_ group).

### Dex prevented H_2_O_2_ induced increase in ROS in AF chondrocytes

**Figure 3:**
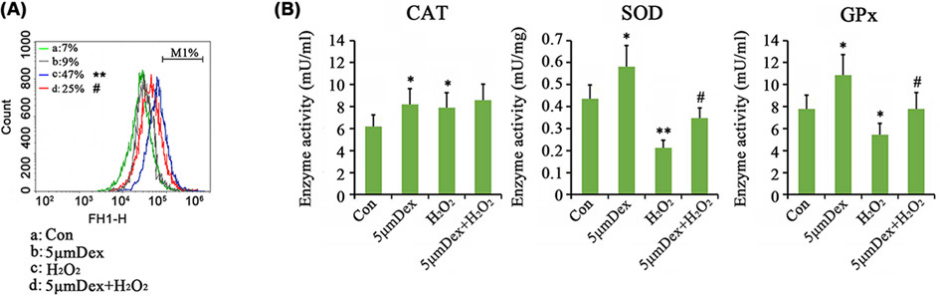
Dex regulated the antioxidative homeostasis in AF chondrocytes Chondrocytes were treated with 5 μM Dex for 24 h. In addition, chondrocytes were treated with 1 mM H_2_O_2_ for 1 h. Afterward, cells were cultured in fresh medium or the medium with supplementation of 5 μM Dex with further incubation for 24 h. Intracellular ROS was monitored using a cell-permeable fluorogenic probe, H2DCFDA by a flow cytometer (**A**). Activities of antioxidative enzymes were tested using the detection kits (**B**). *P<0.05 and **P<0.01 *vs.* control group; ^#^*P*<0.05 *vs.* the group that was treated with H_2_O_2_ alone.

The ROS levels in AF chondrocytes were notably increased by H_2_O_2_ (*P*<0.01, Figure 3A), but only marginally increased by 5 μM Dex. Dex in turn suppressed the H_2_O_2_-mediated increase in ROS levels (*P*<0.05 vs. H_2_O_2_ group). We also tested the activities of antioxidant enzymes including SOD, CAT, and GPx in AF chondrocytes. Dex increased the activities of SOD, CAT, and GPx in AF chondrocytes. H_2_O_2_ increased CAT activity (*P*<0.05, Figure 3B), but significantly decreased SOD (*P*<0.01) and GPx (*P*<0.05) activities in these cells. Compared with treatment with H_2_O_2_ alone, Dex treatment after H_2_O_2_ addition increased the SOD (*P*<0.05 vs. H_2_O_2_ group) and GPx (*P*<0.05 vs. H_2_O_2_ group) activities.

### Dex inhibited the activation of NF-κB and NLRP3 caused by H_2_O_2_

**Figure 4:**
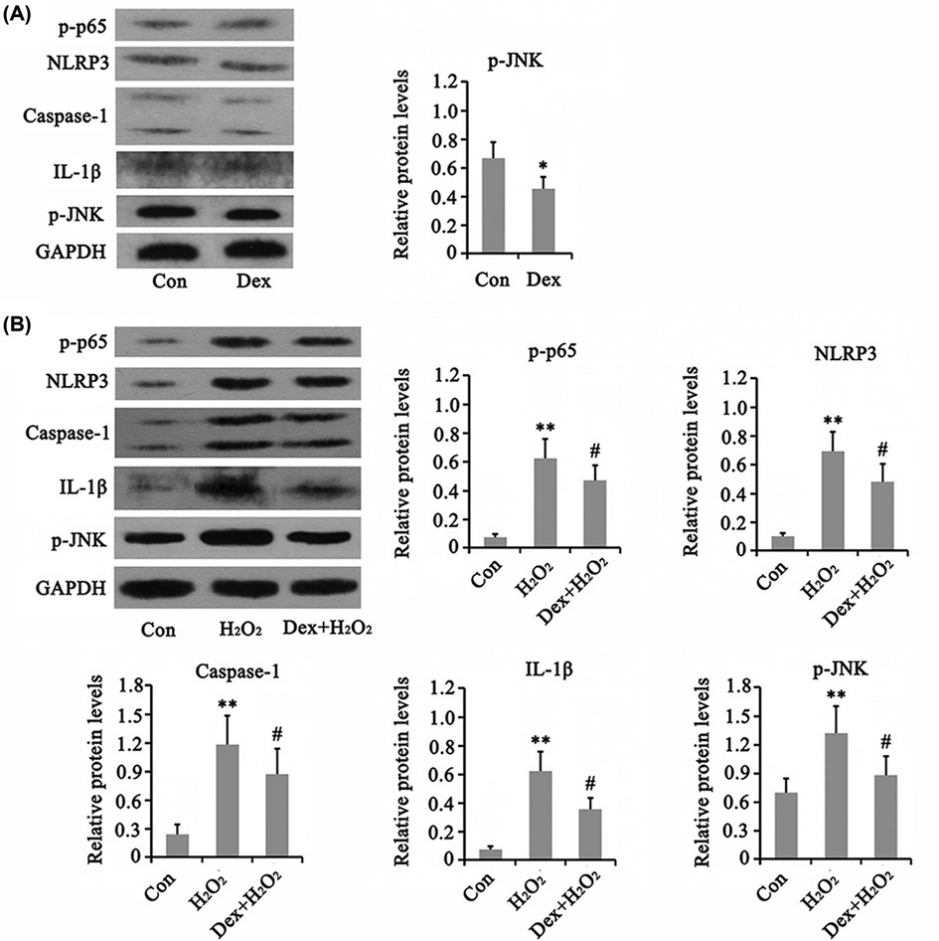
Dex regulated the NF-kB, JNK and NLRP3 pathways in AF chondrocytes (**A**) Chondrocytes were treated with 5 μM Dex for 24 h. Western blot assay was performed to detect the protein levels of p-p65, NLRP3, Caspase-1, IL-1β, and p-JNK. (**B**) Chondrocytes were treated with 1 mM H_2_O_2_ for 1 h. Afterward, cells were cultured in fresh medium or the medium with supplementation of 5 μM Dex with further incubation for 24 h. Western blot assay was performed to detect the protein levels of p-p65, NLRP3, Caspase-1, IL-1β, and p-JNK. *P<0.05 and **P<0.01 *vs.* control group; ^#^*P*<0.05 *vs.* the group that was treated with H_2_O_2_ alone.

As indicated by Western blot analysis, treatment with Dex alone decreased the phosphorylation level of JNK (*P*<0.05, Figure 4), but had no effect on phosphorylation level of p65 and protein levels of NLRP3 and its downstream targets caspase-1 and IL-1β. The phosphorylation level of p65 was increased by H_2_O_2_ (*P*<0.01), but Dex prevented the increase in p65 phosphorylation caused by H_2_O_2_ (*P*<0.05). Furthermore, the protein levels of NLRP3, caspase-1, and IL-1β were also increased when treated with H_2_O_2_ (*P*<0.01), while Dex reversed the increase in these proteins (*P*<0.05). Dex also blocked the increase in JNK phosphorylation induced by H_2_O_2_ (*P*<0.05 vs. H_2_O_2_ group).

**Figure 5:**
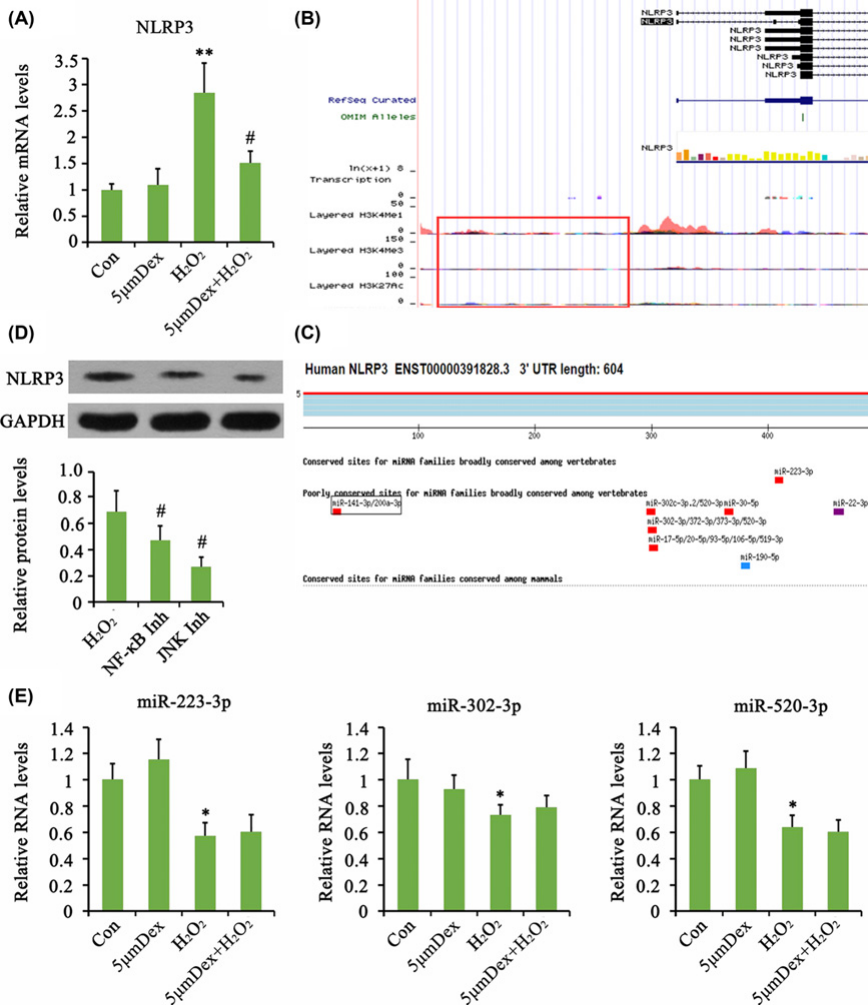
Dex inhibited H_2_O_2_-induced activation of NLRP3 in AF chondrocytes probably through inhibition of NF-κB and JNK pathways Chondrocytes were treated with 5 μM Dex for 24 h. In addition, chondrocytes were treated with 1 mM H_2_O_2_ for 1 h. Afterward, cells were cultured in fresh medium or the medium with the supplementation of 5 μM Dex with further incubation for 24 h. The mRNA level of NLRP3 was evaluated using PCR assay (**A**). Bioinformatics analysis (http://www.genome.ucsc.edu/) indicated that the expression of NLRP3 mRNA was affected by several transcription factors, while it was seldom affected by histone methylation and acetylation (**B**). Bioinformatics analysis (http://www.targetscan.org/vert_72/) indicated that NLRP3 mRNA was targeted by a few miRNAs (**C**). Chondrocytes were treated with 1 mM H_2_O_2_ for 1 h. Afterward, cells were cultured in fresh medium or the medium with the supplementation of tomatidine (10 μM, NF-κB inhibitor) or SP600125 (5 μM, JNK inhibitor) with further incubation for 24 h. The protein levels were evaluated using Western blot assay (**D**). Chondrocytes were treated with 1 mM H_2_O_2_ for 1 h. Afterward, cells were cultured in fresh medium or the medium with the supplementation of 5 μM Dex with further incubation for 24 h. The relative expression levels of indicated mRNA were tested by PCR (**E**). *P<0.05 and **P<0.01 *vs.* control group; ^#^*P*<0.05 *vs.* the group that was treated with H_2_O_2_ alone. NF-κB Inh: administration of NF-κB inhibitor after H_2_O_2_ treatment; JNK Inh: administration of JNK inhibitor after H_2_O_2_ treatment.

We further performed a series of analysis to elucidate the mechanism by which Dex inhibited NLRP3 signaling. PCR analysis showed that NLRP3 mRNA was increased by H_2_O_2_, and this increase was abolished by Dex (*P*<0.01, Figure 5A). This suggested that Dex impaired H_2_O_2_-mediated pre-transcriptional regulation of NLRP3. Bioinformatics analysis (http://www.genome.ucsc.edu/) indicated that the expression of NLRP3 mRNA was affected by several transcription factors (such as AP-1, ΔCREB, NF-κB), while it was seldom affected by histone methylation and acetylation (Figure 5B). The 3′UTR of NLRP3 mRNA was predicted to bind to a few miRNAs, such as miR-223-3p, miR-302-3p, and miR-520-3p (Figure 5C, as indicated by http://www.targetscan.org/vert_72/). Considering that the activity of AP-1 transcription factor is predominantly regulated by JNK signaling pathway, we added a JNK inhibitor to investigate its effect on NLRP3 protein level. Both inhibitors of JNK and NF-κB inhibited the increase in NLRP3 protein level caused by H_2_O_2_ (*P*<0.05, Figure 5D). The expression levels of miR-223-3p, miR-302-3p, and miR-520-3p in AF chondrocytes were evaluated using PCR. Treatment with H_2_O_2_ decreased the expression of these miRNAs (*P*<0.05, Figure 5E), and Dex only marginally increased their expression.

**Figure 6:**
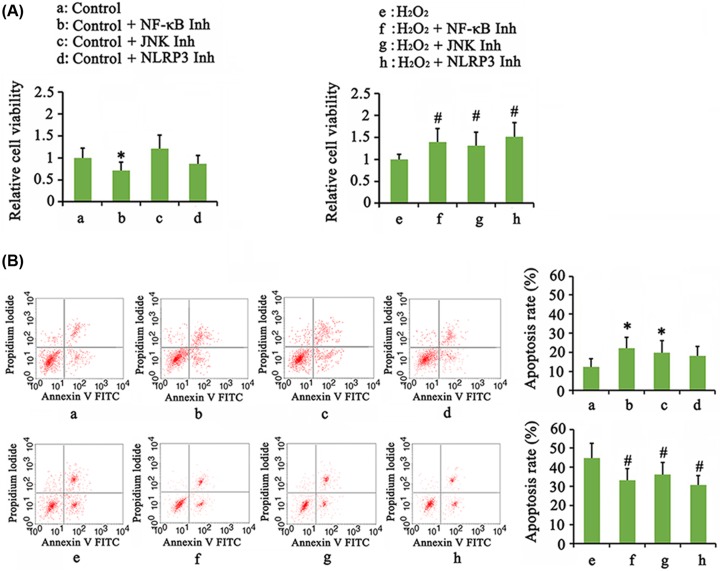
Cell viability and apoptosis rate after the blockage of NF-κB, JNK, and NLRP3 signals Tomatidine (10 μM, NF-κB inhibitor), SP600125 (5 μM, JNK inhibitor) or CY-09 (1 μM, NLRP3 inhibitor) was added to chondrocytes that were treated with 1 mM H_2_O_2_ for 1 h or not. The cell viability (**A**) and apoptosis rate (**B**) were tested after treatments with these inhibitors for 24 h. *P<0.05 *vs.* control group; ^#^*P*<0.05 *vs.* the group that was treated with H_2_O_2_ alone.

**Figure 7:**
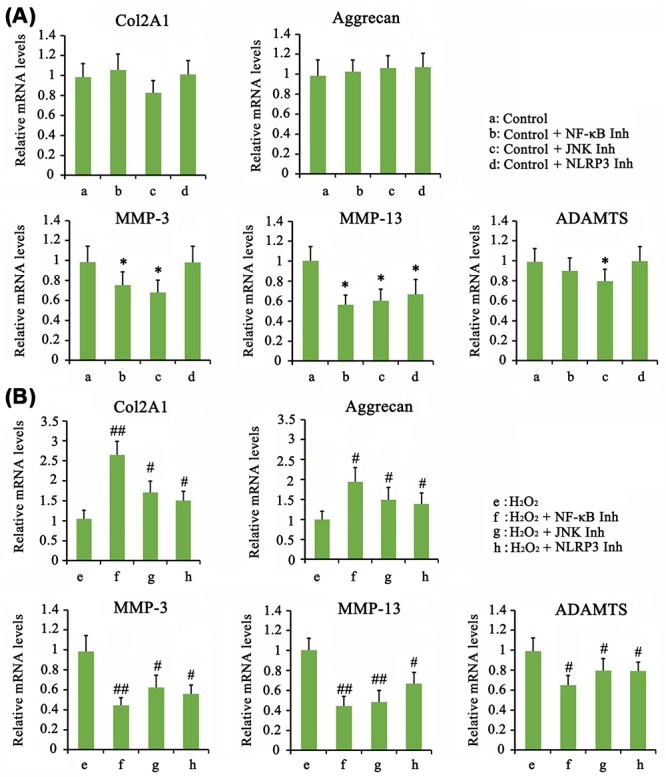
Influence of the degeneration of AF chondrocytes after the blockage of NF-κB, JNK, and NLRP3 signals (**A**) Chondrocytes were treated with Tomatidine (10 μM, NF-κB inhibitor), SP600125 (5 μM, JNK inhibitor) or CY-09 (1 μM, NLRP3 inhibitor) for 24 h. (**B**) In addition, chondrocytes were treated with Tomatidine (10 μM, NF-κB inhibitor), SP600125 (5 μM, JNK inhibitor) or CY-09 (1 μM, NLRP3 inhibitor) for 24 h, followed by treatment with 1 mM H_2_O_2_ for 1 h. PCR was performed to detect the expression of COL2A1, Aggrecan, MMP-3, MMP-13, and ADAMTS1 in AF chondrocytes after treatments with these inhibitors for 24 h. *P<0.05 *vs.* control group; ^#^*P*<0.05, ^##^*P<*0.01 *vs.* the group that was treated with H_2_O_2_ alone.

We found that treatment with NF-κB inhibitor alone suppressed the chondrocyte viability (*P*<0.05, Figure 6A), while inhibitors of JNK and NLRP3 did not significantly affect cell viability. All the inhibition of NF-κB, JNK, and NLRP3 elevated the cell viability that was suppressed by H_2_O_2_ (*P*<0.05, Figure 6A). Inhibitors of NF-κB and JNK increased apoptosis rate (*P*<0.05, Figure 6B), but both of them plus NLRP3 inhibitor lowered the death rate that was increased by H_2_O_2_ (*P*<0.05, Figure 6B). In non-stress condition, inhibition of NF-κB, JNK, and NLRP3 had no effect on expression of COL2A1 and aggrecan mRNA (Figure 7A), but they suppressed expression of MMP-3, MMP-13, or ADAMTS (*P*<0.05). Adding NF-κB, JNK, and NLRP3 inhibitors after H_2_O_2_ treatment increased COL2A1 (*P*<0.05 or *P*<0.01) and aggrecan (*P*<0.05) expression but decreased MMP-3 (*P*<0.05 or *P*<0.01), MMP-13 (*P*<0.05 or *P*<0.01), and ADAMTS (*P*<0.05) compared with treatment with H_2_O_2_ alone (Figure 7B).

### Dex regulated XIAP expression in H_2_O_2_-treated AF chondrocytes

**Figure 8:**
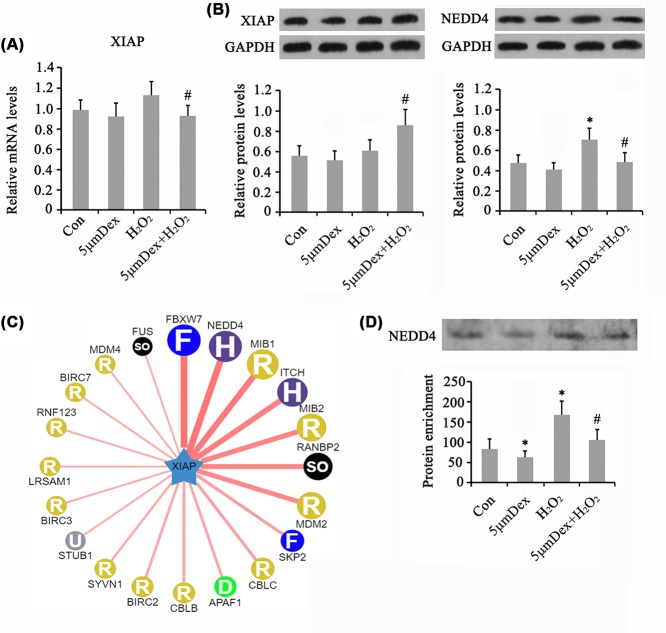
The mechanisms underlying Dex regulating XIAP in AF chondrocytes Chondrocytes were treated with 5 μM Dex for 24 h. In addition, chondrocytes were treated with 1 mM H_2_O_2_ for 1 h. Afterward, cells were cultured in fresh medium or the medium with the supplementation of 5 μM Dex with further incubation for 24 h. The mRNA and protein levels of XIAP were evaluated using PCR (**A**) and Western blot assays (**B**), respectively. Bioinformatics analysis (http://ubibrowser.ncpsb.org/ubibrowser/) showed XIAP protein was regulated by many ubiquitination enzymes (**C**). Immunoprecipitation assay was performed to evaluate the amount of NEDD4 adhering to XIAP (**D**). *P<0.05 *vs.* control group; ^#^*P*<0.05 *vs.* the group that was treated with H_2_O_2_ alone.

Dex had no significant effect on the mRNA level of *XIAP* (Figure 8A). H_2_O_2_ also moderately increased the mRNA level of *XIAP*, while co-treatment with H_2_O_2_ and Dex significantly reduced the *XIAP* mRNA level compared with treatment with H_2_O_2_ alone (*P*<0.05). Western blot analysis showed that XIAP protein level was not changed by treatment with Dex and H_2_O_2_ alone (Figure 8B). Co-treatment with H_2_O_2_ and Dex conversely increased the XIAP protein level compared with treatment with H_2_O_2_ alone (*P*<0.05). Bioinformatics analysis (http://ubibrowser.ncpsb.org/ubibrowser/) showed that XIAP protein was most likely targeted by NEDD4 in addition to FBXW7 (Figure 8C). Both XIAP and NEDD4 were transcriptionally regulated by NF-κB according to bioinformatics analysis. NEDD4 protein level was increased by H_2_O_2_ (*P*<0.05, Figure 8B), while the effect of H_2_O_2_ was attenuated by Dex (*P*<0.05 vs. H_2_O_2_ group). As indicated by the immunoprecipitation assay, the amount of NEDD4 adhering to XIAP was decreased by Dex (*P*<0.05) but increased by H_2_O_2_ (*P*<0.05, Figure 8D). In addition, the increased amount of NEDD4 caused by H_2_O_2_ was attenuated by Dex (*P*<0.05 vs. H_2_O_2_ group).

## Discussion

Dex is commonly used in lumbar discectomy, but its effects on physiological and pathological functions of IVD chondrocytes have never been investigated. Since Dex is an activator of α2-AR and activation of α2-AR by NE is generally associated with rapid cartilage degeneration, it is possible that Dex also accelerates the progression of cartilage degeneration. Under non-stress conditions, Dex indeed decreased the mRNA expression levels of COL2A1 and increased those of MMP-3 and MMP-13 in AF chondrocytes. The reduction in COL2A1 expression is an important hallmark of cartilage degeneration. MMP-3 and MMP-13 are responsible for the decomposition of extracellular matrix, which impairs the cartilage structure and characteristics. Therefore, our results suggested that Dex promoted cartilage degeneration. Nevertheless, the viability of AF chondrocytes was improved by Dex. It has been found that Dex had no effect on cell viability, but presented potential chondrotoxicity at very high dosages (0.175 and 0.25 mg/ml) in a study on articular chondrocytes isolated from healthy equine articular cartilage of the metacarpo/metatarsophalangeal joints [10]. These chondrocytes probably have different characteristics from the chondrocytes isolated from AF tissues in the present study, which could explain the difference in the results. Study herein found that Dex inhibited JNK signal but had no effect on NF-κB and NLRP3 cascades. Treatment with JNK inhibitor increased the apoptosis rate, though decreasing MMP-3 and MMP-13 expression. These data suggest that the effect of Dex on the cartilage degeneration is probably associated with molecular mechanisms independent of JNK signal. Inhibition of JNK under non-stress status probably affects the normal physiological function of chondrocytes, resulting in the increase in apoptosis.

The effect of Dex on AF chondrocytes under non-stress conditions could not be similar to that effect of Dex when it is used during and after lumbar discectomy surgery. Considering that oxidative stress is easily induced during lumbar discectomy, it is more reasonable to investigate the effect of Dex on IVD cartilage under oxidative stress conditions. The present study indicated that Dex did not exert a synergetic or additive effect with H_2_O_2_, but conversely attenuated the detrimental actions of H_2_O_2_. These data were not surprising, since Dex has been confirmed to have antioxidative and anti-inflammatory properties, by numerous previous studies. Sha et al. [7] reported that Dex attenuated lipopolysaccharide-induced liver oxidative stress and cell apoptosis in rats by increasing the activity of GSK-3β/MKP-1/Nrf2 pathway via the α2 adrenergic receptor. An *in vitro* study showed that the oxidative stress induced by H_2_O_2_ was diminished by Dex, thereby preventing the apoptosis of lung alveolar epithelial cells [11]. With respect to chondrocytes, H_2_O_2_ not only induces cell death, but also contributes to its degeneration, because H_2_O_2_ is a strong inducer of ROS production, and ROS is further involved in signaling pathways resulting in chondrocyte degeneration [12]. In the present study, Dex decreased the intracellular levels of ROS partly by enhancing the activities of antioxidant enzymes and thereby, to some extent, attenuating the detrimental effects of H_2_O_2_. An interesting finding in the present study is that H_2_O_2_ increased CAT activity, but decreased SOD and GPx activity. The increase in CAT activity in cells is probably a response to H_2_O_2_, because CAT can directly remove H_2_O_2_. However, a large amount of ROS derived from H_2_O_2_ may impair other antioxidant defenses.

H_2_O_2_-activated inflammatory signals play important roles in the degeneration of chondrocytes. For example, H_2_O_2_ is able to induce the activation of NF-κB signal that can promote the expression and secretion of enzymes such as MMP-3, MMP-13, and ADAMTS [13–15]. In the present study, Dex blocked the activation of NF-κB caused by H_2_O_2_. This outcome is consistent with observations in most previous studies, regarding the anti-inflammatory effect of Dex [13–15]. Therefore, it is possible that Dex protection against H_2_O_2_ is mainly due to its antioxidant activity, which in turn is responsible for NF-κb signal modulation. In addition to NF-κB, the activation of NLRP3 by H_2_O_2_, was also attenuated by Dex treatment. NLRP3 inflammasome is the most well-studied inflammasome that is recognized to play a crucial part in the initiation and continuance of inflammation in various diseases [9,16–18]. Activation of NLRP3 inflammasome is involved in microglial cell activities in hippocampus, thus causing damage in a rat model of traumatic brain injury [9]. The blockage of NLRP3 by Dex administration inhibited microglial activation and increased neuronal viability and cognitive function. Moreover, Dex attenuated pancreatic inflammatory response in mice with pancreatitis by reduction in NLRP3 activation [16]. In lipopolysaccharide-induced acute lung injury, Dex inhibited NLRP3 activation through the up-regulation of miR-381 and miR-381-mediated degeneration of NLRP3 [17]. Lv et al. [18] reported that Dex promotes liver regeneration in mice after 70% partial hepatectomy by suppressing NLRP3 inflammasome and not TLR4/NF-κB, since NLRP3 inhibition is associated with better liver regeneration and liver function recovery, while NF-κB inhibition conversely diminished liver regeneration. In the present study, the inhibition of NLRP3 by Dex occurred likely through the suppression of NF-κB, since NF-κB inhibitor decreased NLRP3 expression and NLRP3-activated caspase-1. We found that the inhibition of JNK signaling was also involved in the mechanism underlying the inhibition of NLRP3 by Dex.

The pathological role of NLRP3 in the degeneration of chondrocytes has also been reported. Activated NLRP3 recruits ASC and caspase-1 to form a protein complex that is essential for caspase-1 activation. Activated caspase-1 leads to the maturation of pre-IL-1β and pre-IL-18. The NLRP3/caspase-1/IL-1β axis has been found to be active in human lumbar cartilaginous endplate degeneration and their expression levels are positively associated with the grades of disc degeneration [19,20]. A study reported that nucleus pulposus cells treated with H_2_O_2_ caused cartilage degeneration as indicated by the changes in expression levels of inflammatory mediators (Interleukin-6, COX-2, and iNOS), major matrix degrading proteases (MMP-3, MMP-13, ADAMTS5, and ADAMTS4), and cartilaginous mark proteins (COL2A1 and SOX9). Honokiol, a low molecular weight natural product, reversed H_2_O_2_-triggered cartilage degeneration, and this protective effect of honokiol is primarily associated with the inhibition of TXNIP/NLRP3/caspase-1/IL-1β signaling axis [21]. However, Bougault et al. [22] suggested that cartilage degeneration does not depend on NLRP3 inflammasome according to the following evidences: (i) osteoarthritis cartilage was not able to produce active IL-1β; (ii) LPS, IL-1α, and TNFα dose-dependently increased MMP-3, MMP-9, and MMP-13 activity in cultured chondrocytes and in NLRP3(−/−) chondrocytes; (iii) these effects of LPS, IL-1α, and TNFα did not change by inhibition of caspase-1 or IL-1β. Although the role of NLRP3 in cartilage degeneration remains controversial, the present study at least confirmed that NLRP3 activation promoted cell death of AF chondrocytes. NLRP3 activation easily triggers pyroptosis and apoptosis by activating caspase family proteins, such as caspase-1 and caspase-3. In the present study, using an inhibitor of NLRP3, we showed that it attenuated H_2_O_2_-triggered cell death.

XIAP is a member of the inhibitor-of-apoptosis proteins (IAP) that represents a family of endogenous caspase inhibitors. Studies have confirmed that up-regulation of XIAP is able to block apoptosis in degenerative nucleus pulposus and osteoarthritic cartilage [23,24]. In addition, XIAP is also involved in the regulation of inflammatory response of cells. Loss of XIAP facilitates the proinflammatory effect of TNF-α and IL-1β, causing severe sterile inflammation in diverse types of cells [25]. Yet, NF-κB acts as an important transcription factor by positively regulating XIAP expression. In many cancer studies, NF-κB-mediated drug resistance is through up-regulation of XIAP [26]. However, herein, treatments with H_2_O_2_ and Dex alone had no effect of both mRNA and protein levels of XIAP. Co-treatment with Dex and H_2_O_2_ caused the reduction in XIAP mRNA level, but the increase in XIAP protein level, compared with treatment with and H_2_O_2_ alone. NEDD4 is an important ubiquitin ligase targeting XIAP. NEDD4-mediated ubiquitination of XIAP can induce the degeneration, thus decreasing total amount of XIAP protein in cells. We found that NEDD4 protein level in cells was increased by H_2_O_2_, but the increase in NEDD4 protein was abolished by Dex. H_2_O_2_-induced the increase in NEDD4 was associated with increased amount of NEDD4 attaching to XIAP, suggesting that NEDD4-mediated ubiquitination of XIAP was enhanced. However, treatment with Dex decreased the amount of NEDD4 attaching to XIAP, which suggested that Dex conferred protective effect from NEDD4-mediated ubiquitination of XIAP.

**Figure 9:**
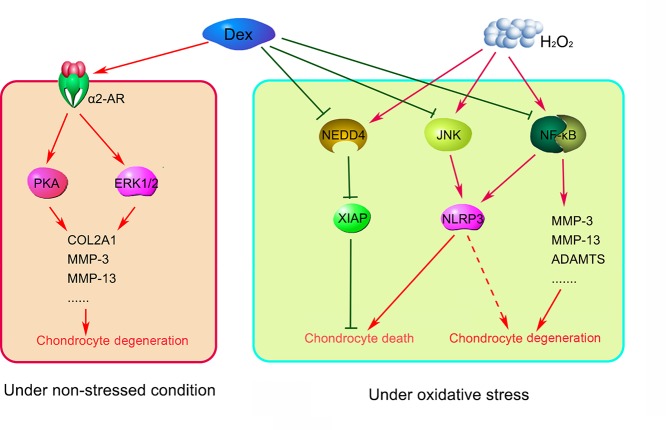
Dex exerts dual effects on the degeneration of human AF chondrocytes depending on the oxidative stress status Under non-stressed condition, Dex induces cartilage degeneration likely through AR-mediated ERK1/2 and PKA pathways according to previous reports [5]. The effect of H_2_O_2_ on cartilage degeneration is partly through NF-κB/NLRP3, JNK/NLRP3 and NEDD4/XIAP pathways. Since, Dex disrupted the effects of H_2_O_2_ on these pathways, Dex prevented the death and degeneration of chondrocytes during oxidative stress. Dashed arrow indicated that the mechanism required further identification.

In summary, the present study demonstrated that Dex treatment is associated with rapid degeneration of chondrocytes under non-stressed conditions. Although both Dex and H_2_O_2_ accelerated the cartilage degeneration, their underlying mechanisms might be different. Dex inducing cartilage degeneration is likely through AR-mediated ERK1/2 and PKA pathways according to previous report [5], while the effect of H_2_O_2_ on cartilage degeneration is more likely through NF-κB/NLRP3, JNK/NLRP3, and NEDD4/XIAP pathways. Since, Dex disrupted the effects of H_2_O_2_ on these pathways, Dex prevented the death and degeneration of chondrocytes during oxidative stress (as indicated in Figure 9). These data suggested a potential protective effect of Dex in lumbar discectomy.

## Abbreviations

AF: annulus fibrosus
AR: adrenoreceptor
CAT: catalase
CCK-8: cell counting kit-8
Dex: Dexmedetomidine
FBS: fetal bovine serum
GPx: glutathione peroxidase
H2DCFDA: 2′,7′-dichlorodihydrofluorescein diacetate
H_2_O_2_: hydrogen peroxide
IVD: intervertebral disc
LBP: lower back pain
NADPH: nicotinamide adenine dinucleotide phosphate
NE: norepinephrine
NEDD4: neural precursor cell expressed developmentally down-regulated protein 4
NLRP3: NACHT, LRR, and PYD domains-containing protein 3
PI: propidium iodide
ROS: reactive oxygen species
SOD: superoxide dismutase
XIAP: X-linked inhibitor-of-apoptosis
